# Down-Regulation of C9orf86 in Human Breast Cancer Cells Inhibits Cell Proliferation, Invasion and Tumor Growth and Correlates with Survival of Breast Cancer Patients

**DOI:** 10.1371/journal.pone.0071764

**Published:** 2013-08-14

**Authors:** Yang-Yang Li, Sha Fu, Xiao-Pai Wang, Hai-Yun Wang, Mu-Sheng Zeng, Jian-Yong Shao

**Affiliations:** 1 State Key Laboratory of Oncology in Southern China, Sun Yat-sen University Cancer Center, Guangzhou, China; 2 Department of Molecular Diagnostics, Sun Yat-sen University Cancer Center, Guangzhou, China; 3 Department of Experiment Research, Sun Yat-sen University Cancer Center, Guangzhou, China; Institute of Molecular and Cell Biology, Biopolis, United States of America

## Abstract

C9orf86 which is a novel subfamily within the Ras superfamily of GTPases, is overexpressed in the majority of primary breast tumors. Few functional studies have focused on the C9orf86 protein; therefore, in this study, we explored the role of C9orf86 in breast carcinogenesis. In our study, we found that silencing of C9orf86 by siRNA in MCF-7 and SK-BR-3 cells resulted in suppressed cell proliferation as well as *in vitro* cell invasion capabilities. Moreover, knockdown of C9orf86 inhibited tumor growth in nude mice. Cell cycle and apoptotic assays showed that the anti-proliferative effect of C9orf86-siRNA was mediated by arresting cells in the G1 phase and promoting apoptosis. In addition, we found that patients with high levels of C9orf86 expression showed a significant trend towards worse survival compared to patients with low C9orf86 expression (P = 0.002). These results provide evidence that C9orf86 represents a novel and clinically useful biomarker for BC patients and plays an important role during the progression of BC.

## Introduction

Breast cancer (BC) is the most frequently diagnosed cancer, and the leading cause of cancer-related deaths in females worldwide, accounting for 23% (1.38 million) of total new cancer cases, and 14% (458,400) of total cancer-related deaths in 2008 [1]. Despite research and resources dedicated to elucidating the molecular mechanisms of breast cancer, the precise mechanisms underlying its initiation and progression remain unclear.

The Ras superfamily is structurally classified into five major branches of small GTPases, including Ras, Rho, Rab, Sar1/Arf, and Ran. Each subfamily of GTPases has distinct roles in the regulation of a variety of cellular processes such as cell proliferation, cell differentiation, apoptosis, survival, cytoskeletal organization, protein transport, and trafficking [2], [3], [4]. In the past three decades, the Ras superfamily of GTPases has become a hot topic in cancer research, as mutant forms of Ras are present in a significant percentage of tumors. For example, high rates of KRAS-activating missense mutations have been detected in non–small cell lung cancer (15 to 20% of tumors) [5], colon adenoma (40%) [6], and pancreatic adenocarcinoma (95%) [7]. RhoB expression is lost in 96% of invasive tumors, and is reduced by 86% in poorly differentiated tumors compared to non-neoplastic epithelium [8]. Rab27B promotes invasive growth and metastasis in estrogen receptor (ER)-positive breast cancer cell lines, and increased expression is associated with poor prognosis in patients [9]. Rab25 is overexpressed in ovarian and breast cancers, which leads to more aggressive forms of cancer [10].

C9orf86 (chromosome 9 open reading frame 86), also known as RBEL1 (Rab-like protein 1), is located at 9q34.3 according to the National Center for Biotechnology Information (NCBI). To date, C9orf86, especially its association with carcinoma, has not been well studied. Functional studies have shown that C9orf86 is a novel subfamily of GTPases within the Ras superfamily. C9orf86 is overexpressed in the majority of primary breast tumors, and knockdown of C9orf86 in MCF-7 breast cancer cells resulted in cell growth suppression associated with apoptosis [11], [12]. These data implicate C9orf86 as a potential oncogene.

To date, the function of C9orf86 in the regulation of carcinogenesis and development of human BC is unclear. Therefore, in this study, we explored the role of C9orf86 in the malignant progression of breast cancer by assaying its function *in vitro* and *in vivo* after C9orf86 knockdown. Furthermore, we analyzed the correlation between C9orf86 protein levels and prognosis as well as clinicopathological characteristics, using immunohistochemistry (IHC) on cancer tissue microrrays (TMAs).

## Results

### C9orf86 is Overexpressed in Human Breast Cancer Cells

**Figure 1:**
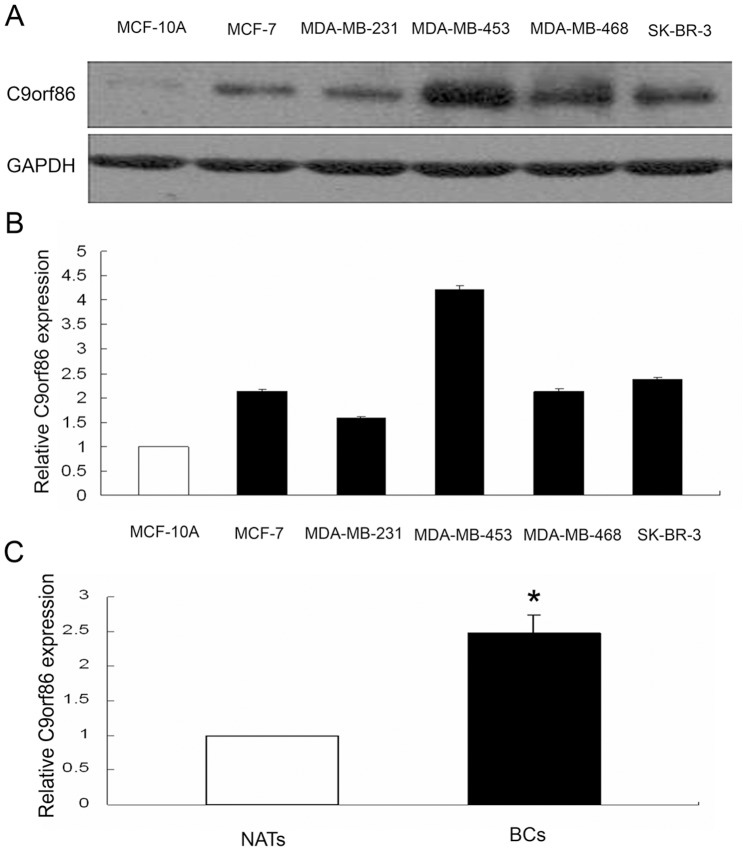
C9orf86 expression in breast cancer cells and tissues. Expression of C9orf86 was quantified in human breast cancer (lanes 2–6), and normal (lane 1) breast epithelial cells by Western blot (A) and qRT-PCR (B). (C) QRT-PCR shows that expression of C9orf86 is increased in invasive BC tissues compared with NATs (P<0.05). Western blotting and RT-PCR were performed using glyceraldehyde-3-phosphate dehydrogenase (GAPDH) as a control.

**Figure 2:**
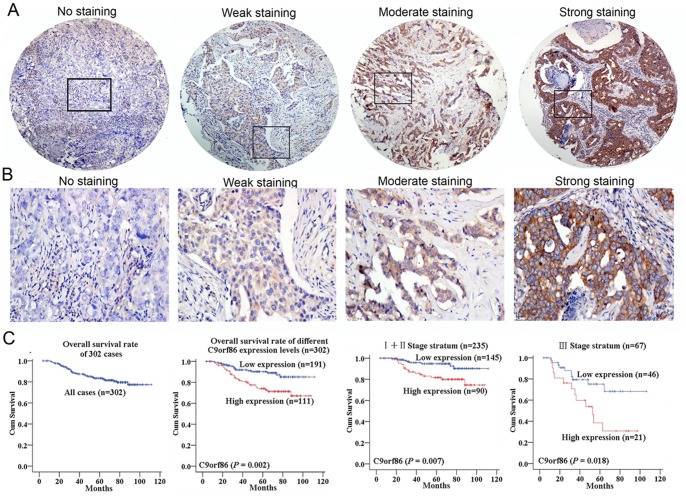
Effect of C9orf86 knockdown on cell proliferation in human breast cancer cells. (A) Forty-eight hours post-transfection, expression of C9orf86 in MCF-7 and SK-BR-3 cells was quantified by western blot analysis. GAPDH was used as a loading control. (B) Colony formation assay. Twenty-four hours post-transfection, MCF-7 and SK-BR-3 cells were seeded into 6-well plates with complete medium and incubated at 37°C for 2 weeks. (C) MTT assay. (D) WST-1 assay. Twenty-four hours post-transfection, MCF-7 and SK-BR-3 cells were seeded into 96-well plates. The colony formation assay (B), MTT assay (C) and WST-1 assay (D) showed that knockdown of C9orf86 in MCF-7 and SK-BR-3 cells resulted in inhibition of cell growth *in vitro*. All data are shown as mean ± SD of triplicate experiments. *P<0.05. NC, negative control.

qRT-PCR and western blot analysis showed that C9orf86 expression was higher in breast cancer cells (MCF-7, MDA-MB-231, MDA-MB-453, MDA-MB-468, and SK-BR-3) than in normal breast epithelial cells (MCF-10A) (Fig. 1A, 1B). Furthermore, C9orf86 was overexpressed in breast cancer tissues, as determined by qRT-PCR and immunohistochemistry (IHC) (Fig. 1C, Fig. 2A and 2B).

### C9orf86 Overexpression Correlates with Poor Prognosis of BC Patients

To investigate C9orf86 protein expression in breast cancer cells, IHC staining was used to detect C9orf86 expression in breast cancer cell biopsies. Strong expression of C9orf86 was found predominantly in the cytoplasm of breast cancer tumor cells (Fig. 2A and 2B). Assessment of survival in the breast cancer patients revealed that patients with high C9orf86 expression showed a significant trend toward worse survival compared to patients with low C9orf86 expression (P = 0.002, Fig. 2C). Further analysis was performed in regard to C9orf86 expression in subsets of breast cancer patients in different clinical stages. The results demonstrated that high C9orf86 expression was also a prognostic factor in patients with stage I, II (P = 0.007) or III (P = 0.018) breast cancer (Fig. 2C).

### C9orf86 Knockdown Results in Cell Growth Suppression

**Figure 3:**
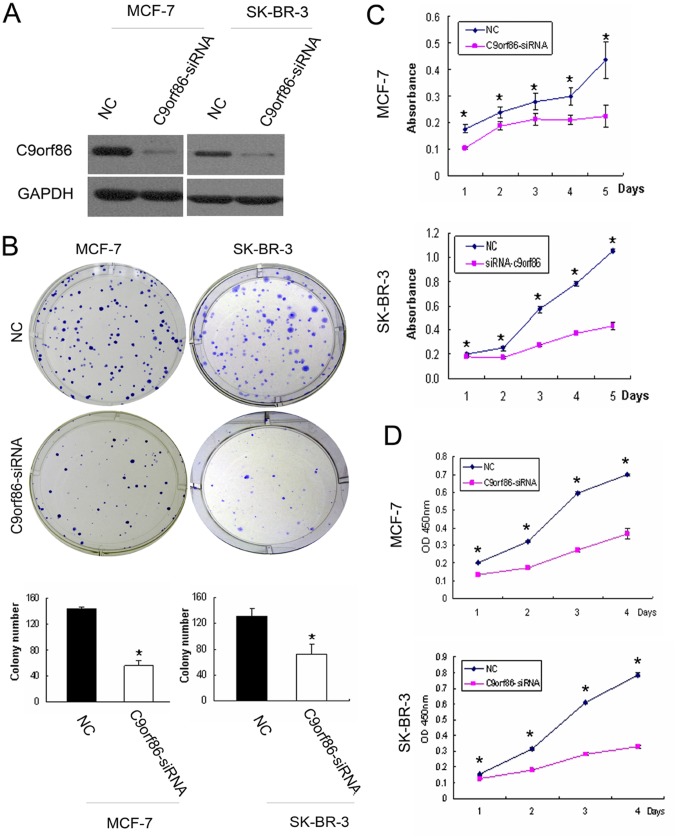
Effect of C9orf86 knockdown on MCF-7 and SK-BR-3 cell growth in nude mice. (A) Photographs of nude mice and tumors extracted from C9orf86 knockdown and NC groups (MCF-7 and SK-BR-3). (B) Tumors were weighed after animals were killed 7 weeks post-tumor cell injection. There was a decreasing trend in both the number of cells and size of tumors in the C9orf86 knockdown and NC group of mice for MCF-7 (P<0.01) and SK-BR-3 (P = 0.261) cells. (C) Growth curves for tumors in MCF-7-C9orf86-siRNA-treated group (n = 6) *versus* the MCF-7-NC-treated group (n = 8) (all P values <0.01) and growth curves for tumors in SK-BR-3-C9orf86-siRNA-treated group (n = 2) *versus* SK-BR-3-NC-treated group (n = 7) (all P values >0.05). (D) The level of C9orf86 mRNA from the tumor 7 weeks after the injection in NC-group was higher than C9orf86-siRNA group (MCF-7 and SK-BR-3, all P values <0.05). Data are shown as the mean ± SD. *P<0.05. NC, negative control.

To address the function of C9orf86 in breast carcinogenesis, we used a siRNA-mediated knockdown approach to suppress the expression of endogenous C9orf86, and subsequently determine the effect on cell growth. Briefly, MCF-7 and SK-BR-3 cells were transfected with C9orf86 siRNA. Immunoblot analysis confirmed knockdown by showing that 72 hours post-transfection, MCF-7 and SK-BR-3 cells transfected with C9orf86-siRNA (50 nM) had decreased C9orf86 protein expression compared to these cells transfected with NC (Fig. 3A). Next, we determined the effect of C9orf86 siRNA on tumor cell proliferation by colony forming, MTT and WST-1 assays. The results showed that knockdown of C9orf86 led to remarkable inhibition of cell growth and proliferation in both MCF-7 and SK-BR-3 cells (Fig. 3B, 3C and 3D).

### Silencing of C9orf86 Suppresses Tumorigenicity *in vivo*

**Figure 4:**
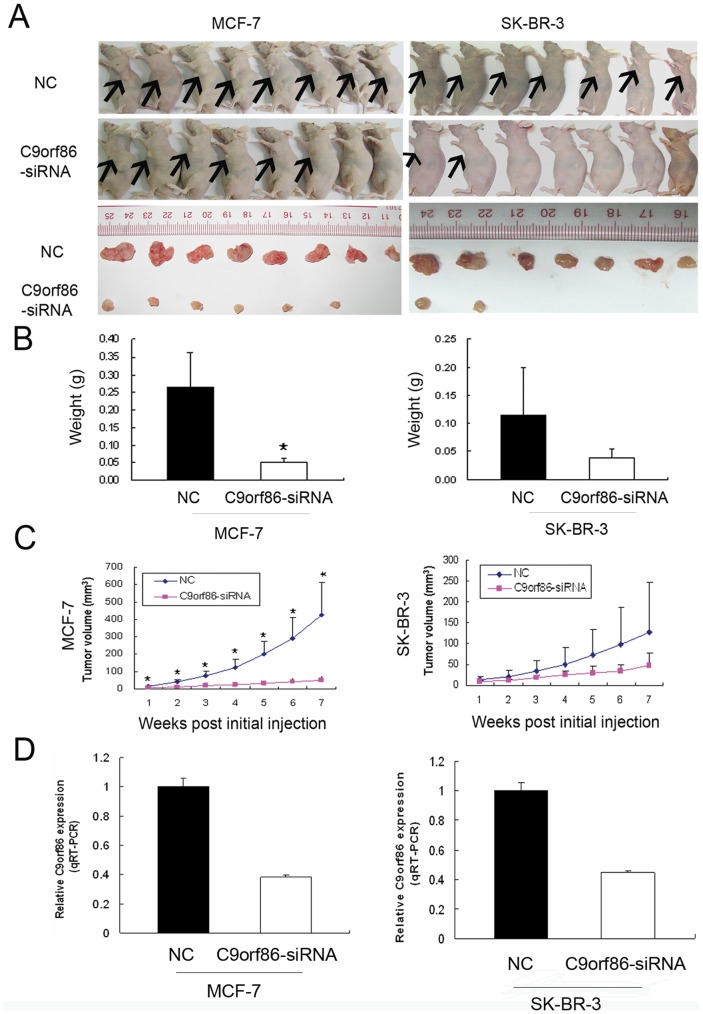
Silencing of C9orf86 induces G1 arrest and apoptosis in breast cancer cells. (A) Cell cycle distribution was analyzed by flow cytometry 72 h after transfection. Bars are shown as the mean ± SD of cells in G1 phase of the cell cycle. (B) Apoptosis was determined by flow cytometric detection of Annexin-V-FITC-positive/PI-negative cells 72 h after infection. Bars are shown as the mean ± SD of cells with Annexin-V-FITC-positive and PI-negative. All data are shown as mean ± SD of two or three independent experiments, *P<0.05. NC, negative control.

To address the potential effects of C9orf86 on the growth of breast cancer cells *in vivo*, MCF-7 or SK-BR-3 cells transfected with C9orf86 siRNA or NC were subcutaneously injected into female nude mice (seven nude mice for SK-BR-3 cells and eight nude mice for MCF-7 cells). As seen in Fig. 4C, tumors rapidly formed in mice injected with MCF-7 (All P values <0.01, Mann-Whitney test) or SK-BR-3 cells transfected with NC, but injection of cells transfected with C9orf86 siRNA led to much lower tumorigenicity. Similarly, compared to the NC group, mice injected with C9orf86 siRNA-transfected cells, showed a significant decrease in both tumor weight and number of MCF-7 (P = 0.002, Mann-Whitney test) and SK-BR-3 cells (P = 0.106, Mann-Whitney test) (Fig. 4B).

### Silencing of C9orf86 Induces G1 Cell Cycle Arrest and Apoptosis *in vitro*

**Figure 5:**
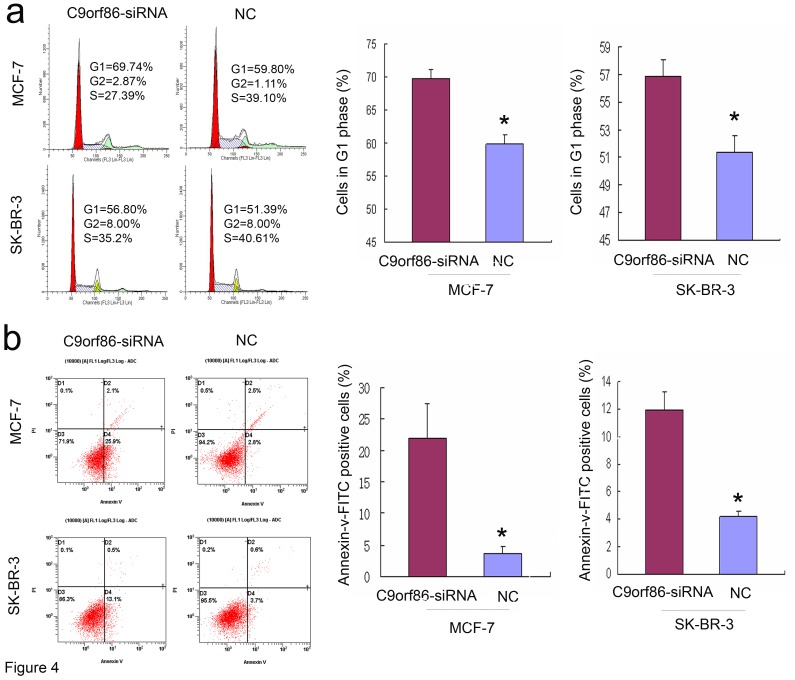
Silencing of C9orf86 expression inhibits invasion ability of MCF-7 cells and SK-BR-3 cells. Cell invasion was assayed in a transwell coated with Matrigel. Cells that crossed the Matrigel-coated filter were fixed, stained, and counted. Six random microscopic fields were counted for each group. The results presented are an average of six random microscopic fields from three independent experiments. Significant reduction of invasion was observed after silencing C9orf86 expression in MCF-7 cells and SK-BR-3 cells. *P<0.05. NC, negative control.

C9orf86 knockdown-mediated suppression of growth could be due to induction of cell cycle arrest or apoptosis. Thus, cell cycle analysis and analysis of apoptosis were performed to identify the mechanisms underlying the observed anti-proliferation effect of C9orf86 siRNA. Cell cycle analysis revealed that the proportion of G1 phase cells increased to 69.74%±1.43% in MCF-7 cells transfected with C9orf86 siRNA compared to 59.80%±1.46% in NC-transfected cells (P = 0.02) (Fig. 5A). The proportion of G1 phase cells increased to 56.80%±1.31% in SK-BR-3 cells transfected with C9orf86-siRNA compared to 51.39%±1.17% in NC-transfected cells (P = 0.001) (Fig. 5a). Flow cytometry was used to assess apoptosis in breast cancer cells after inhibition of C9orf86 using siRNA. Significant differences of Annexin-V-positive apoptotic cells were observed in the C9orf86 siRNA-treated group in comparison to cells transfected with NC. As shown in Fig. 5b, C9orf86 siRNA and NC induced apoptosis in 22.05%±5.44% and 3.6%±1.13% of MCF-7 cells, respectively (P = 0.043). In SK-BR-3 cells, C9orf86 siRNA and NC induced apoptosis in 11.97%±1.27% and 4.17%±0.42%, respectively (P = 0.001). These results suggest that C9orf86 inhibition can induce apoptosis in MCF-7 and SK-BR-3 cells. Furthermore, these data also reveal that knockdown of C9orf86 inhibits cell proliferation by inducing G1 cell cycle arrest and apoptosis.

### Silencing of C9orf86 Inhibits Cell Invasion

**Figure 6:**
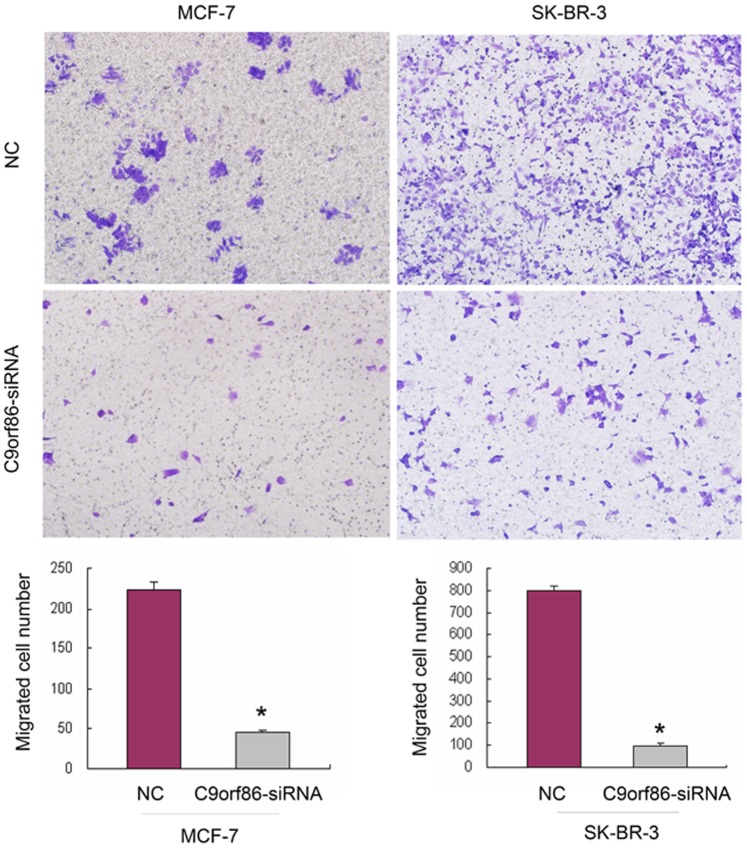
C9orf86 expression and survival in breast cancer patients. (A) and (B) Representative staining of C9orf86 in the cytoplasm of breast cancer cells, by IHC staining at 40× and 200× magnifications. (C) Kaplan-Meier estimates of overall survival curves for different C9orf86 expression levels in breast cancer patients, stratified by clinical stage.

Since cell invasion is a critical property for neoplasm metastasis, we investigated cell invasiveness using *in vitro* invasion assays. Cells that migrated to the bottom of the transwell were fixed, stained, and counted. Matrigel coated transwell chambers were used to evaluate the invasive abilities of the breast cancer cells. Compared to the NC group, C9orf86 siRNA-transfected cells showed a significantly decreased number of migrating MCF-7 and SK-BR-3 cells (Fig. 6). Taken together, these results indicate that silencing of C9orf86 decreases the invasive abilities of breast cancer cells.

## Discussion

The Ras superfamily plays an important role in carcinogenesis and development of cancer, by regulating such processes including cell cycle progression, proliferation, apoptosis, migration, and survival. For example, the R-Ras oncogene promotes tumor cell growth in cervical epithelial cells, and increases their migration potential over collagen through a pathway that involves PI3-K [13]. Rheb controls apoptosis through the FKBP38-dependent regulation of Bcl-2 and Bcl-XL [14]. Rab27A has effects on the invasive and metastatic potentials of breast cancer cells by modulating the secretion of IGF-II, which regulates the expression of p16, VEGF, uPA, cathepsin D, cyclin D1, and MMP-9 [15].

C9orf86 is a novel subfamily within the Ras superfamily. Its role in carcinogenesis has not been well studied and thus remains unclear. Montalbano *et al.* showed that C9orf86 is overexpressed in primary breast cancer [12]. In accordance with this finding, we observed that C9orf86 was overexpressed in breast cancer cell lines and BC tissues. Interestingly, we found that patients with high C9orf86 expression showed a significant trend towards worse survival compared to patients with low C9orf86 expression (P = 0.002). Furthermore, high C9orf86 expression was also a prognostic factor in for patients with stage I, II (P = 0.007) or III (P = 0.018) breast cancer. These results suggest that C9orf86 plays an important role in the development and progression of BC. The anti-proliferation effect of C9orf86-siRNA in MCF-7 and SK-BR-3 cells was also observed in our *in vitro* experiments. Furthermore, we found that C9orf86 siRNA could inhibit tumor growth *in vivo*. To further elucidate the mechanism underlying this anti-proliferative effect, cell cycle and apoptosis analyses were conducted. Interestingly, our results showed that C9orf86 inhibition induced G1 cell cycle arrest and apoptosis in MCF-7 and SK-BR-3 cells, in contrast to a recent report that C9orf86 knockdown-mediated growth suppression was due to the activation of apoptotic signals [11]. Our data suggest that the anti-proliferative effect of C9orf86 siRNA in MCF-7 and SK-BR-3 cells is not only associated with apoptosis, but also correlates with G1 cell cycle arrest.

Both cell migration and invasion are critical properties for the metastasis of cancer cells. Several lines of evidence implicate that the Ras superfamily is involved in cell invasion and tumor metastasis. For example, high expression of RhoC is associated with increased invasion in breast [16], [17], melanoma [18], pancreatic [19], bladder [20], hepatocellular [21], and non–small-cell lung carcinoma [22] primary tumors or cell lines. As a member of Ras subfamily, it was not known whether or not C9orf86 involve in tumor metastasis. To evaluate the effect of C9orf86-siRNA on invasiveness properties of breast cancer cells, invasion assays was conducted. Our results revealed that knockdown of C9orf86 by siRNA led to a significant reduction in the invasive abilities of MCF-7 and SK-BR-3 cells.

Tompkins *et al*. reported that C9orf86 was one of the p14ARF-binding proteins identified by a yeast two-hybrid screen [23]. p14ARF is a tumor suppressor that regulates cell cycle arrest and apoptosis by p53-dependent or p53-independent pathways [24], [25], [26]. However, MCF-7 is a p14ARF-negative cell line [27], [28]. RT-PCR and Western blot analyses confirmed this by demonstrating a lack of p14ARF expression in MCF-7 and SK-BR-3 cells (data not shown). These results indicate that the function of C9orf86 does not correlate with p14ARF in MCF-7 and SK-BR-3 cells. However, the detailed mechanisms underlying C9orf86 knockdown-mediated suppression of cell growth and invasiveness remains to be further investigated.

In conclusion, our results provide valuable information showing that knockdown of C9orf86 in MCF-7 and SK-BR-3 cells has an inhibitory effect on cell growth *in vitro* and *in vivo*, and decreases cell migration *in vivo*. Furthermore, high expression levels of C9orf86 were associated with poor prognosis in patients. Together, these results provide evidence that C9orf86 represents a novel and clinically useful biomarker for BC patients and plays an important role during the progression of BC.

## Materials and Methods

### Patients and Tissue Specimens

BC cases (302) without therapy were randomly selected from January 2000 to December 2002 at Sun Yat-sen University Cancer Center (SYSUCC) and made into TMAs using a MiniCore Tissue Arrayer (Alphelys, Plaisir, France) with a 1-mm needle. Informed consent and clinicopathological information was obtained from all patients in written form. The clinical tumor stage was classified according to the American Joint Committee on Cancer/tumor-lymph node-metastasis classification system [29]. We collected clinicopathological data including patient age, disease stage, tumor size, lymph node metastasis, and recurrence. These data are detailed in Table 1. The institutional Research Medical Ethics Committee of Sun Yat-sen University granted approval for this study (Reference number: B2011-18-01).

**Table 1:** Relationship between C9orf86 expression level and clinicopathological variables of BC patients.

	Number of cases	C9orf86 expression		
Varible		Low	High	*P*-value
**Age (median age)**				
≤47	152	106	46	
>47	150	84	66	0.013
**AJCC clinical stage**				
I+II	235	145	90	
III	67	46	21	0.298
**Lymph node status**				
No metastasis	146	98	48	
Metastasis	156	93	63	0.176
**Recurrence**				
No	247	159	88	
Yes	55	32	23	0.389
**Tumor size**				
≤2.0 cm	78	56	22	
> 2.0, ≤5.0 cm	185	110	75	
>5 cm	23	18	4	0.034

Abbreviations: BC, breast cancer; AJCC, American Joint Committee on Cancer.

### Cell Culture and Knockdown of C9orf86 in MCF-7 and SK-BR-3 Cells

The breast cancer cell lines (MCF-7, MDA-MB-231, MDA-MB-453 and SK-BR-3) and normal nasopharyngeal epithelial cell line (MCF-10A) were purchased from American Type Culture Collection (ATCC, Manassas, USA). MDA-MB-468 breast cancer cell line was kindly provided by Dr. Xiao-Ming Xie (Sun Yat-sen University Cancer Center, Guangzhou, China) [30], [31], [32]. The breast cancer cell lines (MCF-7, MDA-MB-231, MDA-MB-453, MDA-MB-468, and SK-BR-3) were cultured in DMEM with 10% fetal bovine serum (Invitrogen, Carlsbad, CA, USA). The MCF-10A was cultured in KSF (Invitrogen, Carlsbad, CA, USA). All cell lines were grown in a humidified incubator at 37°C with 5% CO_2_.

MCF-7 and SK-BR-3 cells were plated in 6-well dishes at a density of 2×10^5^ cells/well. Knockdown experiments were done 24 h post-seeding. Fifty nanomoles of negative control siRNA (NC) or C9orf86 siRNA (C9orf86-siRNA) duplex oligonucleotides were transfected into cells using Lipofectamine RNAiMAX (Invitrogen, Carlsbad, CA, USA) according to the manufacturer’s instructions. C9orf86-siRNA sequences were 5′-GGCCUAAAGUACCUUCAUATT-3′ (sense) and 5′-UAUGAAGGUACUUUAGGCCTT-3′ (antisense).

### Western Blot Analysis

Cells were digested in SDS lysis buffer containing 50 mmol/L Tris-HCl (pH 7.0), 2% SDS, and 10% glycerol, and incubated for 10 min at 95°C. Fifty micrograms of total cell lysate per lane was separated on 9% SDS-PAGE gels. Mouse mAb of C9orf86 was used for immunoblot analysis (1∶1000 dilution) (Abnova, Taiwan) and mouse mAb of GAPDH (Santa Cruz Biotechnology, CA, USA) was used as a loading control.

### Quantitative Real-time PCR Analysis

Total RNA was extracted from breast cancer cell lines (MCF-7, MDA-MB-231, MDA-MB-453 and SK-BR-3) and normal breast epithelial cell line (MCF-10A) as well as 6 breast tumors and paired adjacent normal tissues (NATs) using TRIzol reagent (Invitrogen, Carlsbad, CA, USA). After reverse transcription of the total RNA, the cDNA was then used as templates for detection of C9orf86 expression by quantitative real-time PCR (qRT-PCR) with the SYBR Green I chemistry. Primers of C9orf86 were used for the PCR reaction with forward 5′-CATCGTGAAGGTTGAAGTCTGG-3′; reverse 5′-GTCCACTGCTTGGTAATGTCG-3′; GAPDH forward 5′-CTGCACCACCAACTGCTTAG-3′; GAPDH reverse 5′-AGGTCCACCACTGACACGTT-3′. Threshold cycles (Ct) for GAPDH (reference) and C9orf86 (sample) were determined in triplicates (shown as arithmetical mean). The quantity of C9orf86 in each BC cell line relative to the average expression in MCF-10A cells, was calculated using the equation: RQ = 2-ΔΔCT.

### Cell Proliferation and Colony Formation Assays

For the MTT cell proliferation assay, 24 h post-transfection, cells were reseeded in 96-well plates at a density of 2×10^3^ cells/well, and incubated overnight in 200 µl culture medium. Twenty-four hours later, cells were stained with 20 µl 3-(4, 5-dimethylthiazol-2-yl)-2, 5-diphenyltetrazolium bromide (MTT) (5 mg/ml), followed by a 4 h incubation at 37°C. After removal of the supernatant, 200 µl dimethyl sulphoxide was added and thoroughly mixed for 15 min. Absorbance was measured at 540 nm using a model 550 microplate reader, with 655 nm as reference filter.

For the WST-1 cell proliferation assay, 24 h post-transfection, cells were reseeded in 96-well plates at a density of 2×10^3^ cells/well, and incubated overnight in 200 µL culture medium. Twenty-four hours later, the cells were washed with PBS and the cell proliferation reagent WST-1 (Roche Molecular Biochemicals, Mannheim, Germany) was added, then samples were incubated for 4 h at 37°C. The absorbance was quantified with a microplate reader (Molecular Devices Corp., CA, USA) at 450 nm.

For colony formation assays, 24 h post-transfection, cells were reseeded in 6-well plates (200 cells per well) and cultured for two weeks. Colonies were fixed with methanol for 10 min, and stained with 1% crystal violet for 20 min. All analyses were performed in triplicate.

### Tumor Growth-promoting Activity of C9orf86 in an Animal Model

Female BALB/c-nude mice (Hunan Slac Jingda Laboratory Animal Co., Ltd., Hunan, China) aged 4–5 weeks, were used for tumor xenografts. The nude mice were then randomly divided into four groups–MCF-7-NC group, MCF-7-C9orf86-siRNA group, SK-BR-3-NC group, and SK-BR-3-C9orf86-siRNA group. Estrogen receptor (ER)-negative SK-BR-3 cells treated with C9orf86-siRNA/NC (100 nM for 48 h) were injected subcutaneously (1×10^7^ cells/tumor) into the left axilla of nude mice in the SK-BR-3-C9orf86-siRNA and SK-BR-3-NC groups. Nude mice in the MCF-7-NC and MCF-7-C9orf86-siRNA groups received β-estradiol (20 mg/kg) intraperitoneally every other day for five times. Ten days after treatment, equivalent amounts of MCF-7 cells treated with C9orf86-siRNA/NC (100 nM for 48 h) were injected subcutaneously (1×10^7^ cells/tumor) into the left axilla of nude mice. Tumor width (W) and length (L) were measured every week. Mice were killed 7 weeks post-injection, and tumors from the four groups were extracted and weighed. Tumor volume was estimated according to the standard formula: V = Π/6×L×W^2^ [33]. All experiments were in accordance with the Ethics Committee for Animal Research of SYSUCC (Reference number: 11020).

### Cell Cycle Assay and Apoptosis Assay

For cell cycle analysis, 72 h post-transfection cells were collected. Then, a total of 1×10^6^ cells were fixed with cold 75% ethanol at 4°C overnight, washed in cold PBS, and stained with propidium iodide (50 ng) containing Rnase (100 ng). The cellular DNA content was quantified using a flow cytometer (BECKMAN COULTER, FULLERTON, CA, USA), and DNA histograms were analyzed using Modifit software (Verity Software House, Lancaster, CA, USA). To identify and quantify apoptotic cells, cells were collected 72 h post-transfection, and then stained with Annexin V-FITC and propidium iodide using the Annexin V-FITC Apoptosis Detection Kit (KEYGEN, China). The percentage of apoptotic cells was quantified using a FACS Calibur Flow cytometer. All analyses were performed in triplicate.

### Invasion Assay

We evaluated the effect of C9orf86-siRNA on invasiveness properties of breast cancer cells using invasion assays. Thirty-six hours post-transfection, MCF-7 or SK-BR-3 cells were detached and resuspended in FBS-free DMEM medium. For the invasion assay, 1×10^5^ cells/200ul FBS-free DMEM medium were plated in the top chamber of the transwell with a matrigel-coated polycarbonate membrane (6.5 mm diameter filters, 8.0 µm pore size; Corning Incorporated, NY, USA). DMEM (500 ul) with 10% FBS was added to the lower chamber as a chemo-attractant. After incubation for 72 h, cells on the lower surface of the membrane were fixed with methanol and stained with 1% crystal violet. Cells that did not migrate through the pores were removed with a cotton swab. Images of invaded cells were acquired using an inverted microscope at a magnification of 200×. The number of invaded cells was counted from five or six fields randomly selected fields.

### Immunohistochemical Staining

IHC staining was performed to examine the expression of C9orf86 in BC tissues. Primary antibody against C9orf86 (1∶300 dilution, Mouse mAb, Abnova) was used in this study. Three observers independently determined consensus scoring of C9orf86 IHC staining using a semi-quantitative estimation [34]. Samples with scores lower than, and equal or more than the median value were considered to be low level expression and high level expression.

### Statistical Analysis

Data was analyzed using SPSS software, version 16.0. Survival curves were plotted by Kaplan-Meier analysis and compared using the log-rank test. Data are expressed as mean ± SD, and the T-test was used to determine the statistically significant differences between the groups. All tests were two-sided, and P<0.05 was considered statistically significant.
